# Nocturnal Activity Is Not Affected by a Long-Duration, Low-Intensity Single Exercise Bout

**DOI:** 10.3390/sports7030056

**Published:** 2019-03-01

**Authors:** Georgia I. Mitrou, Christoforos D. Giannaki, Christina Karatzaferi, Georgios M. Hadjigeorgiou, Eleftherios Lavdas, Maria D. Maridaki, Ioannis Stefanidis, Giorgos K. Sakkas

**Affiliations:** 1 School of PE and Sport Science, University of Thessaly, 42100 Trikala, Greece; gmitrou@pe.uth.gr (G.I.M.); ck@pe.uth.gr (C.K.); gsakkas@med.uth.gr (G.K.S.); 2 Department of Life and Health Sciences, University of Nicosia, Nicosia CY1700, Cyprus; 3 Faculty of Sport, Health and Wellbeing, University of St Mark & St John, Plymouth PL68BH, UK; 4 Medical School, University of Cyprus, Nicosia CY1678, Cyprus; hadjigeorgiou.georgios@ucy.ac.cy; 5 Department of Radiology, University of West Attica, 12210 Athens, Greece; llavdas@teiath.gr; 6 Department of PE and Sport Science, National and Kapodistrian University of Athens, 17237 Athens, Greece; mmarida@phed.uoa.gr; 7 School of Health Science, Department of Medicine, University of Thessaly, 41500 Larissa, Greece; stefanid@med.uth.gr

**Keywords:** sleep, exercise, restlessness, fatigue, periodic limb movements

## Abstract

The aim of the current study was to examine whether prolonged low-intensity aerobic exercise could affect nocturnal activity in healthy individuals. Twenty-one healthy adults (24 ± 3.7 years; 9 females) were enrolled in this study. All participants participated in a 3-h low-intensity walking exercise protocol. Standard biochemical indices were assessed before the exercise protocol and at 72 h. Nocturnal activity and various indices of health were recorded for five consecutive days. The score of muscle pain peaked the night after the exercise protocol (*p* < 0.05) and returned to baseline two days after. No statistical differences were found in any of the parameters examined, including nocturnal activity. Prolonged low-intensity exercise does not affect nocturnal activity. The anecdotal reports suggesting that exercise or/and physical activity could worsen symptoms of motor restlessness during sleep in sleep disorders, such as restless legs syndrome and periodic limb movements, are not supported by this study. However, these findings need to be verified in clinical populations, as well as by using protocols with different forms of exercise.

## 1. Introduction

Research so far has shown that exercise training can be used as a means of prevention or treatment in a wide range of chronic pathological conditions, including cardiovascular, metabolic, pulmonary, and psychiatric diseases [1,2,3]. Additionally, recent studies have shown that exercise helps to manage sleep-related motor disorders, such as restless legs syndrome (RLS) and periodic limb movements during sleep (PLMS) [4,5,6].

Sleep disorders are becoming increasingly common in both the general population and patients with chronic diseases [7,8] and have several adverse effects on physical [9] and mental health [10]. RLS is a sensorimotor disorder characterized by an irresistible urge to move the legs, usually accompanied by an uncomfortable sensation. The symptoms occur mainly during rest/activity and especially in the evening or night, while the symptoms are partially or totally relieved by movement [11]. PLMS are characterized by episodes of stereotyped, repetitive limb movements during sleep [11]. Sleep disturbances and low sleep quality may cause daytime fatigue [12,13] and sleepiness, which also could lead to low productivity [14], human errors, and an increased risk of motor vehicle accidents [15]. On the other hand, it is well known that sleep is a critical contributing factor for recovery following exercise and, thus, may affect exercise performance [16,17]. According to the literature, both acute and chronic exercise could improve sleep quality [18,19].

Evidence revealed that RLS could be associated with increased muscle atrophy (in the case of uremic RLS) [20] and skeletal muscle morphology alternations (in the case of idiopathic RLS) [21], factors which may induce exercise intolerance in this specific group of patients. Even though exercise has been shown to effectively reduce RLS symptoms and PLMS in patients with uremic [4,5,6] and idiopathic RLS [22,23], many patients complain of increased restlessness during sleep especially after intense muscle work or a very long day. This is in line with published guidelines and suggestions such as that of vigorous physical activities and exercise may exacerbate the RLS/PLMS symptoms and thus should be avoided [24], despite the fact that strong evidence supporting this notion is lacking from the literature. 

According to patient anecdotal reports, physical fatigue and intense muscle work disturb sleep by increasing the level of restlessness, thus, affecting the quality and quantity of sleep. Using this as an excuse, patients “are afraid” to participate in any exercise training programs and, therefore, they do not receive any of the numerous benefits of exercise. To the best of our knowledge, there are no studies that have examined whether a prolonged exercise protocol could increase nocturnal activity and worsen sleep quality in both healthy volunteers and patients with sleep disorders. 

On the other hand, recent evidence indicates poor sleep in athletes [25,26]. It seems that this phenomenon is more intense prior to important competitions as athletes suffer from insomnia [27] while restlessness is reported as a potential cause of poor sleep [16,28]. The exercise intensity may also play a role since high-intensity exercise sessions have been followed by poor sleep and sleep disturbance [29,30]. However, we should note that such an association has not been confirmed in other studies [31,32]. Similarly, no changes were observed in sleep parameters after eccentric exercise [33], a form of exercise that is known to induce muscle damage and pain [34]. So far, there are no studies investigating the effect of prolonged mild-intensity exercise or activities equivalent to “a very long day” on nocturnal muscle activity. 

Both creatine kinase (CK) (a marker of muscle damage) and C-reactive protein (CRP) (a marker of inflammation) are widely used in sports and exercise-related research. The concentrations of those biochemical indices appear to be significantly altered post exercise (i.e., after strenuous/muscle-damaging exercise) and could be associated with pain and muscle soreness [35,36]. However, it is still unknown whether those indices could be associated with increased nocturnal activity after prolonged exercise. By investigating the concentrations of those indices, we were able to exclude muscle damage or excessive inflammation as a potential factor that could have affected our findings and that’s why we chose to assess those indices in the current study.

Therefore, the aim of the current study was to examine the effect of three hours of low-intensity continuous walking on nocturnal activity (the activity of limbs assessed during the nocturnal period) in healthy adults prior to a specific study assessing nocturnal activity in a patient group with sleep disorders. 

## 2. Material and Methods

### 2.1. Participants

Twenty-one young adults (12 males/9 females; 24 ± 3.7 years) agreed to voluntarily participate in the present study (Table 1). The study was approved by the ethical committee of the University of Thessaly, Greece, and all participants had to sign an informed consent form prior to enrollment. All volunteers had to be healthy, between 18 and 30 years old and had to avoid any intense activity that would cause muscle damage during the week before beginning the study or during the study. Any prospective participant suffering from orthopedic problems, cardiovascular problems, PLMS, or any other neurological and sleep disorder (checked by health questionnaire) was excluded from the study. In addition, participants working night shifts during the time of the study were also excluded.

### 2.2. Study Design

**Figure 1:**
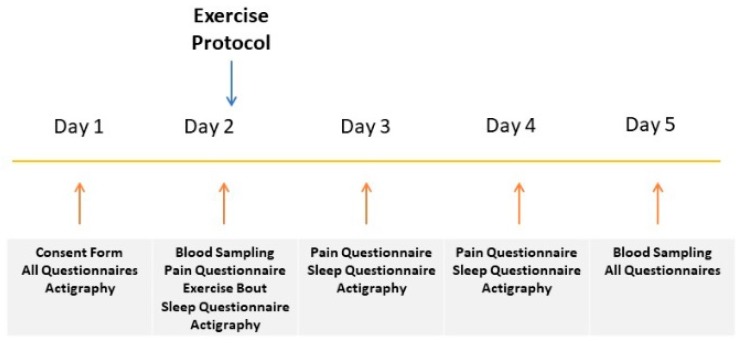
Study Design.

Data were collected for five consecutive days for each participant. During the first day, all participants completed a battery of questionnaires. Some of those questionnaires were also completed on a daily basis. Each participant’s muscle activity during the nocturnal period was recorded at the first night of the study (pre-exercise protocol) and during the next three consecutive nights (after exercise protocol) by an actigraphy system (SOMNOwatch, SOMNOmedics GmbH, Randersacker, Germany). During the second day, all participants engaged in a single bout of low intensity exercise. All participants performed the exercise sessions in the morning at 10 a.m. In addition, they were instructed to keep their usual diet during the study and were instructed to avoid caffeine and alcohol. Before the exercise protocol, a blood sample was collected and again on the fifth day of the study (72 h after the exercise protocol) (Figure 1).

### 2.3. Exercise Protocol

The exercise protocol consisted of three hours of continuous walking on a treadmill with low speed (5 km/h) and zero inclination. This particular protocol was applied in order to simulate the fatigue levels a person can get after a “very long day” of various low-intensity activities [37]. Before starting the exercise protocol, participants had to warm up for 5 min. Heart rate was constantly recorded during the entire protocol using a polar band. During the exercise protocol, the participants were allowed to watch TV or films or listen to music in order to spend their time more pleasantly. 

### 2.4. Biochemical Blood Test

Biochemical examination was performed under fasting conditions at 8:00 a.m. at the ClinLab of the University Hospital under routine procedures including the assessment of C-reactive protein (CRP), urea, creatinine, albumin (ALB), creatine phosphokinase (CPK), total cholesterol, triglycerides (TG), high-density lipoprotein (HDL), low-density lipoprotein (LDL), iron (Fe), and ferritin (FER). 

### 2.5. Questionnaires

Participants were asked to fill in the following standardized and validated questionnaires: Health questionnaire (day 1), SF-36 Quality of Life questionnaire [38] (day 1 and day 5), weekly sleep questionnaire [39] (day 1 and day 5), Epworth sleepiness scale [40] (day 1 and day 5), Pittsburgh sleep quality questionnaire [41] (day 1), daily sleep diary (day 1 to day 5), RLS diagnostic criteria questionnaire [42] (day 1 to day 5), McGill pain questionnaire [43] (day 1 to day 5), and Fatigue Severity Scale questionnaire [44] (day 1 and day 5). 

SF-36 Quality of Life questionnaire is a widely-used tool for assessing the quality of life levels (day 1 and day 5) [38]. The questionnaire consists of 36 questions and has two summary scores: the mental component summary (MCS) and the physical component summary (PCS). The score range is from 0 to 100 with the highest scores indicating better health. Pittsburgh sleep quality questionnaire and a weekly sleep diary (day 1 and day 5) were both used to assess subjective sleep quality. A Pittsburgh sleep quality index (PSQI) score (range 0–21) greater than 5 indicates poor sleep [41]. Daily sleepiness status was measured by the Epworth sleepiness scale (ESS) [40]. The ESS score ranges from 0 to 24 with a score above 10 indicating excessive daily sleepiness. Fatigue levels were assessed using the fatigue severity scale (FSS), which consists of 9 items [44]. Higher scores on the FSS indicate greater fatigue severity. Subjective pain was assessed using the McGill pain questionnaire [43]. The score range is from 0 to 5 with the highest scores indicating more severe pain. Finally, the diagnosis of RLS was made using the four essential criteria of the International RLS Study Group (IRLSSG) [42]. 

### 2.6. Actigraphy

Nocturnal activity was recorded by an actigraphy system (SOMNOwatch, SOMNOmedics GmbH, Randersacker, Germany) during the whole course of the study. The actigraphy system was placed on each participant’s wrist, while an electromyogram was placed on the tibialis anterior muscle. Through the above procedure, we were able to assess both the PLMS index and the isolated limb movements index (ILMV) (total limb movements obtained during the assessment). PLMS is characterized by episodes of stereotyped, repetitive limb movements during sleep [11]. PLMS was scored if it occurred in a series of at least four consecutive movements each lasting 0.5–10 s and separated by intervals of 5–90 s [11]. The number of limb movements that did not meet the criteria for PLMS are described as isolated limb movements. PLMS power–PLMS amplitude are EMG parameters used to calculate various PLMS sub-indices and used for research purposes. All data were analyzed offline in the SOMNOwatch software (SOMNOmedics GmbH, Randersacker, Germany).

### 2.7. Statistics 

The changes from baseline to days 2, 3, 4, and 5 were evaluated using the general linear model repeated measures while chi-square was used for categorical variables. Multiple testing was corrected for in the post hoc analyses using the Bonferroni correction test. The baseline characteristics between males and females were compared using an unpaired t-test. Due to the potential contribution of the variables “age” and “gender” to our findings, those parameters have been added as covariates in the analysis. All statistical analyses were performed using the SPSS version 18.0 (SPSS Inc. Chicago, IL, USA). Data in the text are presented as the mean ± SD and the level for statistical significance was set at *p* ≤ 0.05.

## 3. Results

**Figure 2:**
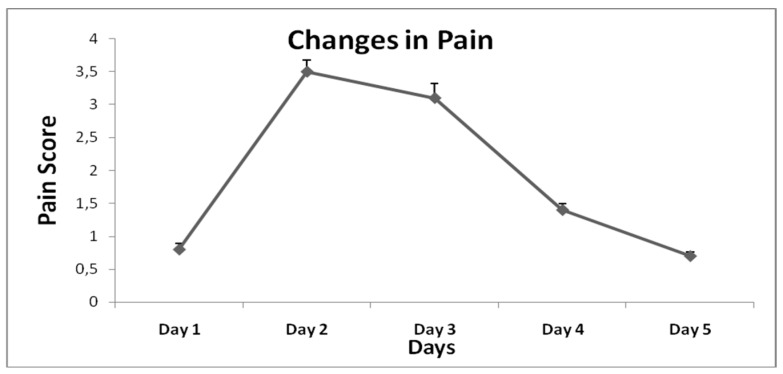
Pain score (mean ± SE) during the course of the study.

The participants’ basic characteristics are presented in Table 1. All blood indices were within the normal range. No differences were found in any of the biochemical indices between day 2 and day 5 (Table 2). Data assessing the nocturnal activity are presented in Table 3. None of the parameters assessed during sleep changed statistically during the study. The changes in the pain score are presented in Figure 2. The score of pain changed significantly during the study. More specifically, statistical differences were found between day 1 and day 2 (0.8 ± 1.8 vs. 3.5 ± 3.6, *p* = 0.047) and between day 2 and day 5 (3.5 ± 3.6 vs. 0.7 ± 1.2, *p* = 0.029). None of the other parameters that were assessed changed during the study course. Heart rate increased gradually during the exercise protocol. As it was expected, significant differences were found between resting heart rate (HR) and HR during the exercise protocol (73.8 ± 2.4 bpm vs. 99.6 ± 2.3 bmp, *p* = 0.000) and between resting HR and HR at the end of the exercise protocol (73.8 ± 2.4 bpm vs. 101.1 ± 2.8 bpm, *p* = 0.000). Gender did not affect the current analysis (*p* > 0.05). The only statistically significant changes were found in the pain score between males and females for day 1 (1.7 ± 2.4 vs. 0.09 ± 0.3, *p* = 0.048), day 2 (5.7 ± 3.8 vs. 1.6 ± 2.4, *p* = 0.010) and day 3 (5.7 ± 5.5 vs. 0.9 ± 1.6, *p* = 0.014) with females declaring higher scores compared to males.

## 4. Discussion

This is the first study to examine whether a long duration, low-intensity walking exercise session could affect nocturnal activity in a healthy population. According to our findings, a 3-h single bout of low-intensity walking exercise did not affect nocturnal activity in healthy volunteers nor did negatively affect subjective sleep quality even though self-reported pain levels were increased immediately after the exercise protocol. It seems that prolonged duration with low-intensity activities can be performed without any adverse negative effects on sleep quality and/or nocturnal activity. 

Previous studies in healthy adults examined the effect of 35 min of vigorous exercise performed two hours before bedtime and found no polysomnographic evidence of disturbed sleep [32]. In addition, a cross-sectional study in adults revealed that evening exercise (including vigorous intensity exercise) is not associated with impaired subjective sleep quality [45]. A recent study in athletes revealed that high-intensity interval training, which constitutes a very popular form of exercise in the last decade, may induce significant impairments in sleep [30], findings that indicate a potential negative role of exercise intensity on sleep.

On the other hand, patients who suffer from movement disorders such as RLS or PLMS, as well as individuals with sleep-related problems, often report that intense muscle work or “a very long day” increases the restlessness during sleep and worsens their sleep quality. This is in line with review articles reporting that vigorous or/and prolonged exercise could exacerbate RLS/PLMS symptoms [24]. Unfortunately, this assumption could act as a significant barrier to exercise and may explain in part the fact that RLS has been reported to be strongly associated among others with low physical activity and exercise capacity [46]. On the other hand, recent studies reveal that adding some vigorous exercise parts in the exercise schedule of both healthy and chronic disease populations could result in further improvements in physical fitness and health-related parameters [47] and, thus, this kind of exercise should be encouraged. In our study, we used an exercise protocol to cause the sensation of a very long, physically active day. The average HR during the 3-hour bout of exercise was 100 beats per minute (50% of HRmax), very similar to the HR recorded in other various daily activities taking place during the day, such as housekeeping activities that produce 95 beats per minute [48]. As a result of our protocol, the self-reported general pain score was significantly increased the day after the protocol (day 2 vs. day 1, *p* = 0.047) and returned to baseline levels after 72 h (day 5 vs. day 1, *p* > 0.05).

Even though this exercise protocol caused an increase in HR and changed the pain score, it did not induce muscle damage since such changes would have affected the biochemical indices that were examined in the current study. We should note that a study which used eccentric exercise in order to induce muscle pain did not observe any changes in sleep-related parameters in healthy young men [33]. On the other hand, our protocol was strong enough to create a sensation of muscle soreness, but this feeling lasted less than 72 h, implying that delayed onset of muscle soreness (DOMS) was avoided [49].

In addition, since long duration exercise causes increased fat oxidation [50], fat was probably the main energy substrate of our participants during the exercise protocol. There is also evidence that increased levels of plasma fatty acids (due to the increased fat oxidation) lead to increased plasma concentrations of tryptophan, causing increases in the 5-hydroxytryptamine (serotonin) brain level [51], which plays an important role in the presence of mental fatigue and sleepiness in humans [51,52] and could increase melatonin secretion [53]. In addition, acute exercise could result in various physiological adaptations, which could beneficially affect sleep including body temperature changes, heart functions (i.e., heart rate), and autonomic function (i.e., heart rate variability), metabolic and central nervous system fatigue [54]. It is possible, therefore, that our exercise protocol could result to sleepiness, something that was evidenced by the fact that the participants spent about 7.4% (not significantly) more time in bed the night after the exercise protocol (day 2). 

Based on our findings, this type of fatigue did not cause significant changes in nocturnal activity even though the isolated leg movements (ILMS), periodic leg movements (PLMS), and position change indices during sleep were increased by 10% (*p* > 0.05) after the exercise protocol and remained increased for three consecutive nights. 

Since healthy volunteers are prone to muscle changes during sleep after a “very long day”, it is reasonable to assume that in patients with RLS or PLMS, a larger change in nocturnal activity could be detected and, therefore, this restlessness would be able to affect sleep duration and quality. However, this assumption remains to be seen with further experiments. We previously showed that both light and heavy intensity exercise performed during the hemodialysis session significantly reduced motor restlessness in patients with uremic RLS [5], while chronic exercise training could significantly reduce RLS symptoms in the same group of patients, improving in parallel subjective sleep quality [4].

In this study, all participants were healthy young adults with normal BMI, normal fatigue and pain scores, and normal quality of life scores. In addition, all participants reported neither sleep problems nor daytime restlessness with any abnormal biochemical indices. It would be interesting to examine whether the same exercise scenario could affect those parameters in chronic disease patients, including patients with sleep disorders and elderly individuals. In addition, it would be interesting to see the effect of exercising at different time-points during the day on the examined variables (i.e., exercising in late evening). 

Unfortunately, in the current study, there are many limitations that need to be acknowledged. The biochemical blood test took place only before the exercise protocol and 72 h after the protocol, missing, thus, the acute and gradual changes in inflammatory indices related to muscle fatigue and damage. The 72 h time point was selected based on a paper published by members of our group [55] showing that 72 h after muscle-damaging exercise training is enough to spot any pick changes in various inflammation and oxidative stress indices. In addition, mental fatigue was not assessed during exercise nor post exercise to see whether the exercise protocol induced mental fatigue. In addition, during the exercise protocol, the level of substrate utilized was not assessed to verify our serotonin hypothesis. Moreover, we did not assess or control the phase of the menstrual cycle of our participants. Finally, the nocturnal activity and the quality and quantity of sleep were not assessed by a full night polysomnography, which is considered the gold standard methodology. The inclusion of polysomnography could allow also examining whether the nocturnal activity levels differ between the different sleep stages. 

## 5. Conclusions

This is the first study to show that a low-intensity long duration aerobic exercise protocol that simulates the sensation of a “very long day” does not affect nocturnal activity in healthy volunteers nor induce any muscle damage. It is evident that nocturnal activity is not solely dependent on the previous muscular activity, but it seems that other more influential factors/conditions such as the presence of RLS or other sleep disorders affect this outcome. It remains to be seen whether a similar protocol would affect restlessness/muscle activity in patients with sleep disorders, such as RLS and PLMS, and whether an exercise training program would improve their restlessness during sleep overall. 

## Figures and Tables

**Table 1:** Participant characteristics.

Variables	Participants
N	21
Gender	12M/9F
Age (years)	24 ± 3.7
BMI (kg/m²)	22.4 ± 2.4
Physical Component Summary (SF36)	81.7 ± 14.4
Mental Component Summary (SF36)	75.6 ± 15.0
Total Score (SF36)	80.3 ± 14.1
Pittsburgh	7.4 ± 5.5
Fatigue Severity Scale	3.2 ± 1.2
RLS	Negative

All data are the mean ± SD. Abbreviations: BMI, body mass index; RLS, restless legs syndrome.

**Table 2:** Biochemical blood test data.

Variables	Day 2	Day 5	*p* values
CRP (mg/L)	0.4 ± 0.3	0.5 ± 0.2	F_1.000, 9.000_ = 6.342, *p* = 0.608
Urea (mg/dl)	26.2 ± 7.0	25.7 ± 5.4	F_1.000, 9.000_ = 0.013, *p* = 0.836
Cr (mg/dl)	0.8 ± 0.1	0.8 ± 0.1	F_1.000, 9.000_ = 1.279, *p* = 0.611
ALB (g/dl)	3.5 ± 0.5	3.3 ± 0.7	F_1.000, 9.000_ = 1.503, *p* = 0.405
CPK (IU/L)	107.9 ± 179.5	88.5 ± 100.9	F_1.000, 9.000_ = 0.375, *p* = 0.731
Total CHO (mg/dl)	124.1 ± 24.6	111.7 ± 29.2	F_1.000, 9.000_ = 1.441, *p* = 0.226
TG (mg/dl)	75.4 ± 24.0	59.9 ± 24.2	F_1.000, 9.000_ = 0.924, *p* = 0.094
HDL (mg/dl)	37.9 ± 10.8	37.0 ± 12.0	F_1.000, 9.000_ = 1.471, *p* = 0.827
LDL (mg/dl)	70.9 ± 17.1	62.7 ± 17.6	F_1.000, 9.000_ = 7.010, *p* = 0.213
HDL/LDL (mg/dl)	0.55 ± 0.18	0.61 ± 0.19	F_1.000, 9.000_ = 0.029, *p* = 0.437
Total CHO/HDL ratio (mg/dl)	3.48 ± 1.0	3.12 ± 0.55	F_1.000, 9.000_ = 0.309, *p* = 0.281
Fe (ug/dl)	61.9 ± 30.4	79.0 ± 26.6	F_1.000, 9.000_ = _0.840_, *p* = 0.123
FER (ng/dl)	66.1 ± 37.8	60.7 ± 30.5	F_1.000, 9.000_ = _0.165_, *p* = 0.679

All data are the mean ± SD. Statistical analyses were performed using the general linear model repeated measures (two time points). Multiple testing was corrected for in the post hoc analyses using the Bonferroni correction test. Abbreviations: CRP, C-reactive protein; Cr, creatinine; ALB, albumin; CPK, creatine phosphokinase; CHO, cholesterol; TG, triglycerides; HDL, high-density lipoprotein; LDL, low-density lipoprotein; Fe, iron; FER, ferritin.

**Table 3:** Activity data.

Variables	Day 1	Day 2	Day 3	Day 4	*p* Values
TIB (in minutes)	423.1 ± 74.8	456.6 ± 65.9	427.3 ± 70.4	441.1 ± 91.2	F_2.820, 47,948_ = 1.1440, *p* = 0.479
ILMS	75.0 ± 39.7	87.7 ± 34.7	84.6 ± 32.4	88.3 ± 44.2	F_2.216, 36.671_ = 0.737, *p* = 0.649
ILMS index (per hour)	10.6 ± 5.1	11.5 ± 4.1	11.8 ± 4.2	11.7 ± 4.8	F_2.358, 40.092_ = 0.339, *p* = 0.833
PLMS	14.9 ± 12.3	17.4 ± 12.8	20.9 ± 26.1	14.7 ± 11.8	F_1.303, 7.817_ = 0.207, *p* = 0.694
PLMS index (per hour)	2.0 ± 1.4	2.3 ± 1.7	3.1 ± 4.3	1.9 ± 1.7	F_1.335, 8.010_ = 0.441, *p* = 0.553
PLMS power [45]	22.8 ± 3.5	21.9 ± 2.0	23.7 ± 3.5	21.7 ± 3.4	F_1.990, 11.939_ = 1.068, *p* = 0.262
PLMS amplitude [45]	70.4 ± 43.5	52.1 ± 21.5	62.8 ± 24.7	55.5 ± 28.1	F_1.509, 9.054_ = 0.047, *p* = 0.332
PLMS duration (s)	2.9 ± 1.6	2.3 ± 0.9	2.5 ± 0.9	2.4 ± 1.0	F_1.613, 9.677_ = 0.073, *p* = 0.480
Position changes	15.8 ± 8.2	18.4 ± 9.2	21.1 ± 9.7	19.6 ± 11.4	F_2.408, 38.521_ = 0.470, *p* = 0.354
Position changes index (per hour)	2.2 ± 1.0	2.4 ± 1.0	2.9 ± 1.4	2.6 ± 1.4	F_2.158, 34.530_ = 0.121, *p* = 0.280

All data are the mean ± SD. Statistical analyses were performed using the general linear model repeated measures (four time points). Multiple testing was corrected for in the post hoc analyses using the Bonferroni correction test. Abbreviations: TIB, time in bed; ILMS, isolated limb movements in sleep; PLMS, periodic limb movements in sleep.
